# Supplementary material for: What are the barriers and facilitators to polio vaccination and eradication programs? A systematic review
Source: PLOS Glob Public Health. 2022 Nov 16;2(11):e0001283. doi: 10.1371/journal.pgph.0001283 (PMC10022167; doi:10.1371/journal.pgph.0001283)
Supplement: S2 Data — (DOCX) [file pgph.0001283.s002.docx]

***S2 Data: Search Strings***

**Barriers and Facilitators to Polio Vaccination and Eradication Programs: A Systematic Review**

**After Deduplication:** September 18, 2020 (2332 results)

**OVID Medline:** September 15, 2020 (1206 results)

Imported September 18, 2020 (1208 results)

| Ovid MEDLINE: Epub Ahead of Print, In-Process & Other Non-Indexed Citations, Ovid MEDLINE® Daily and Ovid MEDLINE® <1946-Present> | | | |
| --- | --- | --- | --- |
| **#** | **Search Statement** | **Results** | **Annotation** |
| 1 | exp Poliomyelitis/pc [Prevention & Control] | 5046 |  |
| 2 | polio*.tw,kf. | 31615 |  |
| 3 | 1 or 2 | 32244 |  |
| 4 | Disease Eradication/ | 2801 |  |
| 5 | Immunization Programs/ | 10896 |  |
| 6 | exp Poliovirus Vaccines/ | 7487 |  |
| 7 | (polio* adj3 (vaccin* or immuniz* or immunis* or eradicat* or eliminat* or program* or campaign*)).tw,kf. | 8652 |  |
| 8 | 4 or 5 or 6 or 7 | 23037 |  |
| 9 | implementation science/ | 484 |  |
| 10 | Health Behavior/ | 50343 |  |
| 11 | Health Knowledge, Attitudes, Practice/ | 112120 |  |
| 12 | health education/ or health promotion/ | 128116 |  |
| 13 | (implement* or adopt* or intervention* or scaling or scale-up or knowledge or attitude* or belief* or compliance or accept* or education or promotion or barrier* or facilitat*).tw,kf. | 3831310 |  |
| 14 | 9 or 10 or 11 or 12 or 13 | 3923991 |  |
| 15 | 3 and 8 and 14 | 1832 |  |
| 16 | limit 15 to (english language and humans and journal article) | 1399 |  |
| 17 | limit 16 to "review" | 190 |  |
| 18 | limit 16 to "systematic review" | 13 |  |
| 19 | 16 not (17 or 18) | 1206 |  |

**OVID Embase:** September 15, 2020 (1312 results)

Imported September 15, 2020 (1312 results)

| Embase Classic+Embase <1947 to 2020 September 14> | | | |
| --- | --- | --- | --- |
| **#** | **Search Statement** | **Results** | **Annotation** |
| 1 | poliomyelitis/pc [Prevention] | 6506 |  |
| 2 | polio*.tw,kw. | 39842 |  |
| 3 | 1 or 2 | 42009 |  |
| 4 | disease eradication/ | 2850 |  |
| 5 | preventive health service/ | 29042 |  |
| 6 | poliomyelitis vaccine/ | 10733 |  |
| 7 | (polio* adj3 (vaccin* or immuniz* or immunis* or eradicat* or eliminat* or program* or campaign*)).tw,kw. | 10431 |  |
| 8 | 4 or 5 or 6 or 7 | 46787 |  |
| 9 | implementation science/ | 1283 |  |
| 10 | health behavior/ | 66953 |  |
| 11 | attitude to health/ | 115198 |  |
| 12 | health education/ or exp health promotion/ | 191427 |  |
| 13 | (implement* or adopt* or intervention* or scaling or scale-up or knowledge or attitude* or belief* or compliance or accept* or education or promotion or barrier* or facilitat*).tw,kw. | 5039467 |  |
| 14 | 9 or 10 or 11 or 12 or 13 | 5179277 |  |
| 15 | 3 and 8 and 14 | 2394 |  |
| 16 | limit 15 to (human and english language) | 1862 |  |
| 17 | limit 16 to article | 1312 |  |

**EBSCO CINAHL:** September 15, 2020 (516 results)

Imported September 18, 2020 (518 results)

| **#** | **Query** | **Limiters/Expanders** | **Results** |
| --- | --- | --- | --- |
| S18 | S3 AND S8 AND S15 | Expanders - Apply equivalent subjects  Narrow by Source types: - academic journals  Narrow by Language: - english  Search modes - Boolean/Phrase | 516 |
| S17 | S3 AND S8 AND S15 | Expanders - Apply equivalent subjects  Narrow by Language: - english  Search modes - Boolean/Phrase | 599 |
| S16 | S3 AND S8 AND S15 | Expanders - Apply equivalent subjects  Search modes - Boolean/Phrase | 613 |
| S15 | S9 OR S10 OR S11 OR S12 OR S13 OR S14 | Expanders - Apply equivalent subjects  Search modes - Boolean/Phrase | 1,858,563 |
| S14 | (implement* or adopt* or intervention* or scaling or scale-up or knowledge or attitude* or belief* or compliance or accept* or education or promotion or barrier* or facilitat*) | Expanders - Apply equivalent subjects  Search modes - Boolean/Phrase | 1,841,307 |
| S13 | (MH "Health Promotion") | Expanders - Apply equivalent subjects  Search modes - Boolean/Phrase | 66,597 |
| S12 | (MH "Health Education") | Expanders - Apply equivalent subjects  Search modes - Boolean/Phrase | 28,067 |
| S11 | (MH "Health Knowledge") OR (MH "Attitude to Health") | Expanders - Apply equivalent subjects  Search modes - Boolean/Phrase | 72,800 |
| S10 | (MH "Health Behavior") | Expanders - Apply equivalent subjects  Search modes - Boolean/Phrase | 49,636 |
| S9 | (MH "Implementation Science") | Expanders - Apply equivalent subjects  Search modes - Boolean/Phrase | 210 |
| S8 | S4 OR S5 OR S6 OR S7 | Expanders - Apply equivalent subjects  Search modes - Boolean/Phrase | 8,607 |
| S7 | (polio* N3 (vaccin* or immuniz* or immunis* or eradicat* or eliminat* or program* or campaign*)) | Expanders - Apply equivalent subjects  Search modes - Boolean/Phrase | 2,624 |
| S6 | (MH "Poliovirus Vaccine+") | Expanders - Apply equivalent subjects  Search modes - Boolean/Phrase | 1,818 |
| S5 | (MH "Immunization Programs") | Expanders - Apply equivalent subjects  Search modes - Boolean/Phrase | 5,650 |
| S4 | (MH "Disease Eradication") | Expanders - Apply equivalent subjects  Search modes - Boolean/Phrase | 1,176 |
| S3 | S1 OR S2 | Expanders - Apply equivalent subjects  Search modes - Boolean/Phrase | 5,256 |

**Web of Science:** September 15, 2020 (1105 results)

Imported September 18, 2020 (1107 results)

(TS=(polio* NEAR/3 (vaccin* OR immuniz* OR immunis* OR eradicat* OR eliminat* OR program* OR campaign*))

AND

TS=(implement* OR adopt* OR intervention* OR scaling OR scale-up OR knowledge OR attitude* OR belief* OR compliance OR accept* OR education OR promotion OR barrier* OR facilitat*))

AND

LANGUAGE:(English) AND DOCUMENT TYPES:(Article)
