# Supplementary material for: What are the barriers and facilitators to polio vaccination and eradication programs? A systematic review
Source: PLOS Glob Public Health. 2022 Nov 16;2(11):e0001283. doi: 10.1371/journal.pgph.0001283 (PMC10022167; doi:10.1371/journal.pgph.0001283)
Supplement: S3 Data — (DOCX) [file pgph.0001283.s003.docx]

***S3 Data: MMAT/CASP Assessment***

**Bedford et al., 2017**

CASP Table

| **Author/Year** | **Article Name** | **Screening Questions (**Yes/No/Can’t tell) | | **Methodological Quality Criteria** (Yes/No/Can’t tell) | |
| --- | --- | --- | --- | --- | --- |
| **Bedford et al., 2017** | Community Engagement in Liberia: Routine Immunization Post-Ebola | 1. Was there a clear statement of the aim of the research? | Yes (in abstract) | 3. Was the research design appropriate to address the aims of the research? | Yes |
|  |  |  |  | 4.Was the recruitment strategy appropriate to the aims of the research? | Yes |
|  |  | 2. Do the collected data allow to address the research questions? | Yes | 5. Was the data collected in a way that addressed the research issue? | Yes |
|  |  |  |  | 6. Has the relationship between researcher and participants been adequately considered? | Yes |
|  |  |  |  | 7. Have ethical issues been taken into considerations? | Yes |
|  |  |  |  | 8. Was the data analysis sufficiently rigorous? | Yes |
|  |  |  |  | 9. Is there a clear statement of findings? | Yes |
|  |  |  |  | 10. How valuable is the research? | Valuable |

**Chincholikar et al., 2000**

MMAT

| **Author/Year** | **Article Name** | **Screening Questions (**Yes/No/Can’t tell) | | **Methodological Quality Criteria** (Yes/No/Can’t tell) | |
| --- | --- | --- | --- | --- | --- |
| Chincholikar et al., 2000 | Evaluation of Pulse-Polio Immunisation in Rural Area of Maharashtra | 1. Are there clear research questions? | Yes | 3. Is there an adequate rationale for using a mixed methods design to address the research question? | Yes |
|  |  |  |  | 4. Are the different components of the study effectively integrated to answer the research question? | Yes |
|  |  | 2. Do the collected data allow to address the research questions? | Yes | 5. Are the outputs of the integration of qualitative and quantitative components adequately interpreted? | Yes |
|  |  |  |  | 6. Are there divergence and inconsistencies between quantitative and qualitative results adequately addressed? | No |
|  |  |  |  | 7. Do the different components of the study adhere to the quality criteria of each tradition of the methods involved? | Yes |

**Closser et al., 2014**

MMAT

| **Author/Year** | **Article Name** | **Screening Questions (**Yes/No/Can’t tell) | | **Methodological Quality Criteria** (Yes/No/Can’t tell) | |
| --- | --- | --- | --- | --- | --- |
| Closser et al. (2014) | The Impact of Polio Eradication on Routine Immunization and Primary Health Care: A Mixed-Methods Study | 1. Are there clear research questions? | Yes | 3. Is there an adequate rationale for using a mixed methods design to address the research question? | Yes |
|  |  |  |  | 4. Are the different components of the study effectively integrated to answer the research question? | Yes |
|  |  | 2. Do the collected data allow to address the research questions? | Yes | 5. Are the outputs of the integration of qualitative and quantitative components adequately interpreted? | Yes |
|  |  |  |  | 6. Are there divergence and inconsistencies between quantitative and qualitative results adequately addressed? | No |
|  |  |  |  | 7. Do the different components of the study adhere to the quality criteria of each tradition of the methods involved? | Yes |

**Closser et al. 2016**

CASP Table

| **Author/Year** | **Article Name** | **Screening Questions (**Yes/No/Can’t tell) | | **Methodological Quality Criteria** (Yes/No/Can’t tell) | |
| --- | --- | --- | --- | --- | --- |
| **Closser et al. 2016** | The Global Context of Vaccine Refusal: Insights from a Systematic Comparative Ethnography of the Global Polio Eradication Initiative | 1. Was there a clear statement of the aim of the research? | Yes | 3. Was the research design appropriate to address the aims of the research? | Yes |
|  |  |  |  | 4.Was the recruitment strategy appropriate to the aims of the research? | Yes |
|  |  | 2. Do the collected data allow to address the research questions? | Yes | 5. Was the data collected in a way that addressed the research issue? | Yes |
|  |  |  |  | 6. Has the relationship between researcher and participants been adequately considered? | Yes |
|  |  |  |  | 7. Have ethical issues been taken into considerations? | Can’t tell |
|  |  |  |  | 8. Was the data analysis sufficiently rigorous? | Yes |
|  |  |  |  | 9. Is there a clear statement of findings? | Yes |
|  |  |  |  | 10. How valuable is the research? | Valuable |

**Cohart et al., 1962**

MMAT

| **Author/Year** | **Article Name** | **Screening Questions (**Yes/No/Can’t tell) | | **Methodological Quality Criteria** (Yes/No/Can’t tell) | |
| --- | --- | --- | --- | --- | --- |
| Closser et al. (2014) | The Impact of Polio Eradication on Routine Immunization and Primary Health Care: A Mixed-Methods Study | 1. Are there clear research questions? | Yes | 3. Is there an adequate rationale for using a mixed methods design to address the research question? | Yes |
|  |  |  |  | 4. Are the different components of the study effectively integrated to answer the research question? | Yes |
|  |  | 2. Do the collected data allow to address the research questions? | Yes | 5. Are the outputs of the integration of qualitative and quantitative components adequately interpreted? | No |
|  |  |  |  | 6. Are there divergence and inconsistencies between quantitative and qualitative results adequately addressed? | No |
|  |  |  |  | 7. Do the different components of the study adhere to the quality criteria of each tradition of the methods involved? | Yes |

**Habib et al., 2017**

MMAT

| **Author/Year** | **Article Name** | **Screening Questions (**Yes/No/Can’t tell) | | **Methodological Quality Criteria** (Yes/No/Can’t tell) | |
| --- | --- | --- | --- | --- | --- |
| Habib et al., 2017 | Knowledge and perceptions of polio and polio immunization in polio high-risk areas of Pakistan | 1. Are there clear research questions? | Yes | 3. Is there an adequate rationale for using a mixed methods design to address the research question? | Yes |
|  |  |  |  | 4. Are the different components of the study effectively integrated to answer the research question? | Yes |
|  |  | 2. Do the collected data allow to address the research questions? | Yes | 5. Are the outputs of the integration of qualitative and quantitative components adequately interpreted? | Yes |
|  |  |  |  | 6. Are there divergence and inconsistencies between quantitative and qualitative results adequately addressed? | No |
|  |  |  |  | 7. Do the different components of the study adhere to the quality criteria of each tradition of the methods involved? | Yes |

**Haq et al., 2019**

CASP Table

| **Author/Year** | **Article Name** | **Screening Questions (**Yes/No/Can’t tell) | | **Methodological Quality Criteria** (Yes/No/Can’t tell) | |
| --- | --- | --- | --- | --- | --- |
| Haq et al., 2019 | System within systems: challenges and opportunities for the Expanded Programme on Immunisation in Pakistan | 1. Was there a clear statement of the aim of the research? | Yes | 3. Was the research design appropriate to address the aims of the research? | Yes |
|  |  |  |  | 4.Was the recruitment strategy appropriate to the aims of the research? | Yes |
|  |  | 2. Do the collected data allow to address the research questions? | Yes | 5. Was the data collected in a way that addressed the research issue? | Yes |
|  |  |  |  | 6. Has the relationship between researcher and participants been adequately considered? | Can’t tell |
|  |  |  |  | 7. Have ethical issues been taken into considerations? | Yes |
|  |  |  |  | 8. Was the data analysis sufficiently rigorous? | Yes |
|  |  |  |  | 9. Is there a clear statement of findings? | Yes |
|  |  |  |  | 10. How valuable is the research? | Valuable |

**Hussain et al., 2015**

CASP Table

| **Author/Year** | **Article Name** | **Screening Questions (**Yes/No/Can’t tell) | | **Methodological Quality Criteria** (Yes/No/Can’t tell) | |
| --- | --- | --- | --- | --- | --- |
| Hussain et al., 2015 | Partition and Poliomyelitis: An Investigation of the Polio Disparity Affecting Muslims during India's Eradication Program | 1. Was there a clear statement of the aim of the research? | Yes | 3. Was the research design appropriate to address the aims of the research? | Yes |
|  |  |  |  | 4.Was the recruitment strategy appropriate to the aims of the research? | Yes |
|  |  | 2. Do the collected data allow to address the research questions? | Yes | 5. Was the data collected in a way that addressed the research issue? | Yes |
|  |  |  |  | 6. Has the relationship between researcher and participants been adequately considered? | Yes |
|  |  |  |  | 7. Have ethical issues been taken into considerations? | Yes |
|  |  |  |  | 8. Was the data analysis sufficiently rigorous? | Yes |
|  |  |  |  | 9. Is there a clear statement of findings? | Yes |
|  |  |  |  | 10. How valuable is the research? | Valuable |

**Ianni et al., 1960**

MMAT

| **Author/Year** | **Article Name** | **Screening Questions (**Yes/No/Can’t tell) | | **Methodological Quality Criteria** (Yes/No/Can’t tell) | |
| --- | --- | --- | --- | --- | --- |
| Ianni et al., 1960 | Age, social, and demographic factors in acceptance of Polio vaccination | 1. Are there clear research questions? | Yes | 3. Is there an adequate rationale for using a mixed methods design to address the research question? | Yes |
|  |  |  |  | 4. Are the different components of the study effectively integrated to answer the research question? | Can’t tell |
|  |  | 2. Do the collected data allow to address the research questions? | Yes | 5. Are the outputs of the integration of qualitative and quantitative components adequately interpreted? | Yes |
|  |  |  |  | 6. Are there divergence and inconsistencies between quantitative and qualitative results adequately addressed? | Can’t tell |
|  |  |  |  | 7. Do the different components of the study adhere to the quality criteria of each tradition of the methods involved? | Yes |

**Khan et al., 2016**

CASP Table

| **Author/Year** | **Article Name** | **Screening Questions (**Yes/No/Can’t tell) | | **Methodological Quality Criteria** (Yes/No/Can’t tell) | |
| --- | --- | --- | --- | --- | --- |
| Khan et al., 2016 | Challenges to health workers and their opinions about parents’ refusal of oral polio vaccination in the Khyber Pakhtoon Khawa (KPK) province, Pakistan | 1. Was there a clear statement of the aim of the research? | Yes | 3. Was the research design appropriate to address the aims of the research? | Yes |
|  |  |  |  | 4.Was the recruitment strategy appropriate to the aims of the research? | Yes |
|  |  | 2. Do the collected data allow to address the research questions? | Yes | 5. Was the data collected in a way that addressed the research issue? | Yes |
|  |  |  |  | 6. Has the relationship between researcher and participants been adequately considered? | Yes |
|  |  |  |  | 7. Have ethical issues been taken into considerations? | Yes |
|  |  |  |  | 8. Was the data analysis sufficiently rigorous? | Yes |
|  |  |  |  | 9. Is there a clear statement of findings? | Yes |
|  |  |  |  | 10. How valuable is the research? | Valuable |

**Murele et al., 2014**

CASP Table

| **Author/Year** | **Article Name** | **Screening Questions (**Yes/No/Can’t tell) | | **Methodological Quality Criteria** (Yes/No/Can’t tell) | |
| --- | --- | --- | --- | --- | --- |
| Murele et al., 2014 | Vaccine perception among acceptors and non-acceptors in Sokoto State, Nigeria | 1. Was there a clear statement of the aim of the research? | Yes | 3. Was the research design appropriate to address the aims of the research? | Yes |
|  |  |  |  | 4.Was the recruitment strategy appropriate to the aims of the research? | Yes |
|  |  | 2. Do the collected data allow to address the research questions? | Yes | 5. Was the data collected in a way that addressed the research issue? | Yes |
|  |  |  |  | 6. Has the relationship between researcher and participants been adequately considered? | No |
|  |  |  |  | 7. Have ethical issues been taken into considerations? | No |
|  |  |  |  | 8. Was the data analysis sufficiently rigorous? | Yes |
|  |  |  |  | 9. Is there a clear statement of findings? | Yes |
|  |  |  |  | 10. How valuable is the research? | Valuable |

**Ndiaye et al., 2003**

MMAT

| **Author/Year** | **Article Name** | **Screening Questions (**Yes/No/Can’t tell) | | **Methodological Quality Criteria** (Yes/No/Can’t tell) | |
| --- | --- | --- | --- | --- | --- |
| Ndiaye et al., 2003 | The value of community participation in disease surveillance: a case study from Niger | 1. Are there clear research questions? | Yes | 3. Is there an adequate rationale for using a mixed methods design to address the research question? | Yes |
|  |  |  |  | 4. Are the different components of the study effectively integrated to answer the research question? | Yes |
|  |  | 2. Do the collected data allow to address the research questions? | Yes | 5. Are the outputs of the integration of qualitative and quantitative components adequately interpreted? | Yes |
|  |  |  |  | 6. Are there divergence and inconsistencies between quantitative and qualitative results adequately addressed? | No |
|  |  |  |  | 7. Do the different components of the study adhere to the quality criteria of each tradition of the methods involved? | Yes |

**Nuwaha et al., 2000**

CASP Table

| **Author/Year** | **Article Name** | **Screening Questions (**Yes/No/Can’t tell) | | **Methodological Quality Criteria** (Yes/No/Can’t tell) | |
| --- | --- | --- | --- | --- | --- |
| Nuwaha et al., 2000 | Causes of low attendance at National Immunization Days for polio eradication in Bushenyi District, Uganda | 1. Was there a clear statement of the aim of the research? | Yes | 3. Was the research design appropriate to address the aims of the research? | Yes |
|  |  |  |  | 4.Was the recruitment strategy appropriate to the aims of the research? | Yes |
|  |  | 2. Do the collected data allow to address the research questions? | Yes | 5. Was the data collected in a way that addressed the research issue? | Yes |
|  |  |  |  | 6. Has the relationship between researcher and participants been adequately considered? | Yes |
|  |  |  |  | 7. Have ethical issues been taken into considerations? | Yes |
|  |  |  |  | 8. Was the data analysis sufficiently rigorous? | Yes |
|  |  |  |  | 9. Is there a clear statement of findings? | Yes |
|  |  |  |  | 10. How valuable is the research? | Valuable |

**Pervaiz et al., 2017**

MMAT

| **Author/Year** | **Article Name** | **Screening Questions (**Yes/No/Can’t tell) | | **Methodological Quality Criteria** (Yes/No/Can’t tell) | |
| --- | --- | --- | --- | --- | --- |
| Pervaiz et al., 2017 | Fractional-Dose inactivated poliovirus vaccine campaign Sindh Province, Pakistan, 2016 | 1. Are there clear research questions? | Yes | 3. Is there an adequate rationale for using a mixed methods design to address the research question? | Yes |
|  |  |  |  | 4. Are the different components of the study effectively integrated to answer the research question? | Yes |
|  |  | 2. Do the collected data allow to address the research questions? | Yes | 5. Are the outputs of the integration of qualitative and quantitative components adequately interpreted? | Yes |
|  |  |  |  | 6. Are there divergence and inconsistencies between quantitative and qualitative results adequately addressed? | Can’t tell |
|  |  |  |  | 7. Do the different components of the study adhere to the quality criteria of each tradition of the methods involved? | Yes |

**Shah et al., 2019**

CASP Table

| **Author/Year** | **Article Name** | **Screening Questions (**Yes/No/Can’t tell) | | **Methodological Quality Criteria** (Yes/No/Can’t tell) | |
| --- | --- | --- | --- | --- | --- |
| Shah et al., 2019 | “This is Pakhtun disease”: Pakhtun health journalists’ perceptions of the barriers and facilitators to polio vaccine acceptance among the high-risk Pakhtun community in Pakistan | 1. Was there a clear statement of the aim of the research? | Yes | 3. Was the research design appropriate to address the aims of the research? | Yes |
|  |  |  |  | 4.Was the recruitment strategy appropriate to the aims of the research? | Yes |
|  |  | 2. Do the collected data allow to address the research questions? | Yes | 5. Was the data collected in a way that addressed the research issue? | Yes |
|  |  |  |  | 6. Has the relationship between researcher and participants been adequately considered? | Yes |
|  |  |  |  | 7. Have ethical issues been taken into considerations? | Yes |
|  |  |  |  | 8. Was the data analysis sufficiently rigorous? | Yes |
|  |  |  |  | 9. Is there a clear statement of findings? | Yes |
|  |  |  |  | 10. How valuable is the research? | Valuable |

**Simpson et al., 2014**

MMAT

| **Author/Year** | **Article Name** | **Screening Questions (**Yes/No/Can’t tell) | | **Methodological Quality Criteria** (Yes/No/Can’t tell) | |
| --- | --- | --- | --- | --- | --- |
| Simpson et al., 2014 | Polio eradication initiative in Afghanistan 1997-2013 | 1. Are there clear research questions? | Yes | 3. Is there an adequate rationale for using a mixed methods design to address the research question? | Yes |
|  |  |  |  | 4. Are the different components of the study effectively integrated to answer the research question? | Yes |
|  |  | 2. Do the collected data allow to address the research questions? | Yes | 5. Are the outputs of the integration of qualitative and quantitative components adequately interpreted? | Yes |
|  |  |  |  | 6. Are there divergence and inconsistencies between quantitative and qualitative results adequately addressed? | No |
|  |  |  |  | 7. Do the different components of the study adhere to the quality criteria of each tradition of the methods involved? | Yes |

**Varghese et al., 2012**

CASP Table

| **Author/Year** | **Article Name** | **Screening Questions (**Yes/No/Can’t tell) | | **Methodological Quality Criteria** (Yes/No/Can’t tell) | |
| --- | --- | --- | --- | --- | --- |
| Varghese et al./2012 | The interactions of ethical notions and moral values of immediate stakeholders of immunisation services in two Indian states: a qualitative study | 1. Was there a clear statement of the aim of the research? | Yes | 3. Was the research design appropriate to address the aims of the research? | Yes |
|  |  |  |  | 4.Was the recruitment strategy appropriate to the aims of the research? | Yes |
|  |  | 2. Do the collected data allow to address the research questions? | Yes | 5. Was the data collected in a way that addressed the research issue? | Yes |
|  |  |  |  | 6. Has the relationship between researcher and participants been adequately considered? | Can’t tell |
|  |  |  |  | 7. Have ethical issues been taken into considerations? | Yes |
|  |  |  |  | 8. Was the data analysis sufficiently rigorous? | Yes |
|  |  |  |  | 9. Is there a clear statement of findings? | Yes |
|  |  |  |  | 10. How valuable is the research? | Valuable |

**Varghese et al., 2014**

CASP Table

| **Author/Year** | **Article Name** | **Screening Questions (**Yes/No/Can’t tell) | | **Methodological Quality Criteria** (Yes/No/Can’t tell) | |
| --- | --- | --- | --- | --- | --- |
| Varghese et al., 2014 | Advancing the application of systems thinking in health: understanding the growing complexity governing immunization services in Kerala, India | 1. Was there a clear statement of the aim of the research? | Yes | 3. Was the research design appropriate to address the aims of the research? | Yes |
|  |  |  |  | 4.Was the recruitment strategy appropriate to the aims of the research? | Yes |
|  |  | 2. Do the collected data allow to address the research questions? | Yes | 5. Was the data collected in a way that addressed the research issue? | Yes |
|  |  |  |  | 6. Has the relationship between researcher and participants been adequately considered? | Yes |
|  |  |  |  | 7. Have ethical issues been taken into considerations? | Yes |
|  |  |  |  | 8. Was the data analysis sufficiently rigorous? | Yes |
|  |  |  |  | 9. Is there a clear statement of findings? | Yes |
|  |  |  |  | 10. How valuable is the research? | Valuable |

**Kishore et al., 2003**

CASP Table

| **Author/Year** | **Article Name** | **Screening Questions (**Yes/No/Can’t tell) | | **Methodological Quality Criteria** (Yes/No/Can’t tell) | |
| --- | --- | --- | --- | --- | --- |
| Kishore et al., 2003 | Qualitative study of wild polio cases in high-risk districts of Uttar Pradesh, India. | 1. Was there a clear statement of the aim of the research? | Yes | 3. Was the research design appropriate to address the aims of the research? | Yes |
|  |  |  |  | 4.Was the recruitment strategy appropriate to the aims of the research? | Yes |
|  |  | 2. Do the collected data allow to address the research questions? | Yes | 5. Was the data collected in a way that addressed the research issue? | Yes |
|  |  |  |  | 6. Has the relationship between researcher and participants been adequately considered? | No |
|  |  |  |  | 7. Have ethical issues been taken into considerations? | No |
|  |  |  |  | 8. Was the data analysis sufficiently rigorous? | No |
|  |  |  |  | 9. Is there a clear statement of findings? | Yes |
|  |  |  |  | 10. How valuable is the research? | Valuable |

**Bisrat et al., 2013**

CASP Table

| **Author/Year** | **Article Name** | **Screening Questions (**Yes/No/Can’t tell) | | **Methodological Quality Criteria** (Yes/No/Can’t tell) | |
| --- | --- | --- | --- | --- | --- |
| Bisrat et al., 2013 | Cross-border wild polio virus transmission in CORE Group Polio Project areas in Ethiopia. | 1. Was there a clear statement of the aim of the research? | Yes | 3. Was the research design appropriate to address the aims of the research? | Yes |
|  |  |  |  | 4.Was the recruitment strategy appropriate to the aims of the research? | Yes |
|  |  | 2. Do the collected data allow to address the research questions? | Yes | 5. Was the data collected in a way that addressed the research issue? | Yes |
|  |  |  |  | 6. Has the relationship between researcher and participants been adequately considered? | Yes |
|  |  |  |  | 7. Have ethical issues been taken into considerations? | Yes |
|  |  |  |  | 8. Was the data analysis sufficiently rigorous? | Yes |
|  |  |  |  | 9. Is there a clear statement of findings? | Yes |
|  |  |  |  | 10. How valuable is the research? | Valuable |

***S3 Table: Study characteristics***

| **Study characteristics** | | | | | | | |
| --- | --- | --- | --- | --- | --- | --- | --- |
| # | Study title | Author names | Year of publication | Country of study | Type of study (quantitative or mixed-methods) | Population/subgroup | Study objective |
| 1 | Community engagement in Liberia: Routine immunization post-Ebola | Juliet Bedford, Ketan Chitnis, et al. | 2017 | Liberia | Qualitative (focus group discussions & interviews) | Community members, primary caregivers, and community leaders | To provide an overview of the community engagement and social mobilization activities that were conducted and reports the key findings of a rapid qualitative assessment conducted immediately after the campaign that focused on community perceptions of routine immunization in the post-Ebola context |
| 2 | Evaluation of pulse-polio immunisation in rural area of Maharashtra | S.V Chincholikar, and R.D Prayag | 2000 | India | Mixed-methods (evaluation survey &I interviews) | Rural and urban population | To carry out an evaluation survey on Pulse-polio immunisation in rural areas of Maharashtra to assess the immunisation coverage, the knowledge regarding pulse-polio and the routine immunisation schedule |
| 3 | The impact of polio eradication on routine immunization and primary health care: a mixed-methods study | Svea Closser, Kelly Cox, et al. | 2014 | Ethiopia, Nigeria, Rwanda, Angola, India, Nepal, and Pakistan | Mixed-methods (global cross-national time series analysis & participant observation, and semi-structured interviews | Community members, ground-level staff, district and national leadership | To quantitatively evaluate the effects of the initial scale-up of polio eradication activities, and the number of polio vaccination campaigns per year on measures of RI and PHC. Also qualitatively examined potential mediators of these relationships in 8 case studies |
| 4 | The global context of vaccine refusal: insights from a systematic comparative ethnography of the Global Polio Eradication Initiative | Svea Closser, Anat Rosenthal, et al. | 2016 | Ethiopia, Nigeria, Rwanda, Angola, India, Nepal, and Pakistan | Qualitative (document review, participant observation, and interviews) | National health officials from the Ministry of Health, WHO and UNICEF, district-level health officials, ground-level community health workers, parents of children targeted during polio campaign | Describes an ethnographic study of the GPEI examining polio vaccine acceptance and refusal to understand how global phenomena shape local behaviour |
| 5 | Factors influencing the acceptance of oral poliovirus vaccine | Edward M. Cohart, Colin White, et al. | 1962 | United States of America | Mixed-methods (interviews) | General population (households with children) | To study the community’s knowledge of and attitudes to oral vaccine, its knowledge of, attitudes to, and previous acceptance of Salk-type vaccines, and the relationship of these factors to acceptance of oral vaccine |
| 6 | Knowledge and perceptions of polio and polio immunization in polio high-risk areas of Pakistan | Muhammad Atif Habib, Sajid Bashir Soofi, et al. | 2017 | Pakistan | Mixed-methods (cross-sectional survey, focus group discussions, in-depth interviews) | Mothers and elderly women, community stakeholders, male decision makers, polio program staff, religious leaders, health care providers, political leaders | To identify knowledge, attitudes, an practices of target populations about polio vaccine and its eradication, and to estimate coverage of routine immunization and oral polio vaccine |
| 7 | System within systems: challenges and opportunities for the Expanded Programme on Immunisation in Pakistan | Zaeem Haq, Babar Tasneem Shaikh, et al. | 2019 | Pakistan | Qualitative (semi-structured interviews) | Key informants from various tiers of immunisation policy and programme implementation | To explore the EPI’s insights about its structural and implementation arrangements within the larger governance system, and the ensuing challenges as well as opportunities |
| 8 | Partition and Poliomyelitis: an investigation of the Polio disparity affecting Muslims during India’s Eradication Program | Rashid S. Hussain, Stephen T McGarvey, et al. | 2015 | India | Qualitative (participant observation, historical document research, and interviews) | Vaccination teams, Global Polio Eradication Initiative (GPEI) stakeholders and families with vaccine-eligible children | To describe how the socio-political and historical context of Muslim populations in India shaped the polio disparity |
| 9 | Age, social, and demographic factors in acceptance of Polio vaccination | Francis A.J. Ianna, Robert Albrecht, et al. | 1960 | United States of America | Quantitative (interviews) | Households with vaccine-eligible children | To provide basic data for estimates of the level of polio vaccination by age grades in the State population, and to obtain information on the comparative demographic and social characteristics of vaccinated and unvaccinated groups |
| 10 | Challenges to health workers and their opinions about parents’ refusal of oral polio vaccination in the Khyber Pakhtoon Khawa (KPK) province, Pakistan | Tahir Mehmood Khan, and Muhammad Umar Sahibzada | 2016 | Pakistan | Qualitative (focus group discussions) | Health workers | To explore the challenges faced by health workers during the polio health campaign and identifying the factors causing parents to refuse oral polio vaccination |
| 11 | Vaccine perception among acceptors and non-acceptors in Sokoto State, Nigeria | Bola Murele, Rui Vaz, et al. | 2014 | Nigeria | Qualitative (interviews) | Caregivers, opinion and religious leaders, community leaders | Explore and document the perceptions of  vaccine among caregivers in Sokoto State, who accept or refuse the immunization of their children against polio virus |
| 12 | The value of community participation in disease surveillance: a case study from Niger | Serigne M. Ndiaye, Linda Quick, et al. | 2003 | Niger | Mixed-methods (printed resources, interviews, participant observation) | Epidemiologists, health care workers, community members | To assess the social and cultural factors that affect the detection and reporting of disease cases in a surveillance system, using acute flaccid paralysis (AFP) surveillance in Niger as a case study |
| 13 | Causes of low attendance at National Immunization Days for polio eradication in Bushenye District, Uganda | F. Nuwaha, G. Mulindwa | 2000 | Uganda | Qualitative (focus group discussions) | Sample from general population | To investigate the factors influencing attendance for polio National Immunization Days (NIDs) using the Attitudes Social influence self Efficacy model as a guiding theoretical framework |
| 14 | Fractional-dose inactivated poliovirus vaccine campaign - Sindh Province, Pakistan, 2016 | A. Pervaiz, C. Mbaeyi, et al. | 2017 | Pakistan | Mixed-methods (interviews) | caregivers | To describe the findings of an assessment preparatory activities and subsequent implementation of the polio campaign |
| 15 | “This is a Pakhtun disease”: Pakhtun health journalists’ perceptions of the barriers and facilitators to polio vaccine acceptance among the high-risk Pakhtun community in Pakistan | Sayyed Shah, Tamar Ginossar, et al. | 2019 | Pakistan | Qualitative (interviews) | Health journalists | To understand Pakhtun journalists’ perceptions of the barriers to and facilitators for OPV in their communities, including their own beliefs regarding polio and OPV |
| 16 | Polio eradication initiative in Afghanistan, 1997-2013 | Diane Simpson, Nahad Sadr-Azedi, et al. | 2014 | Afghanistan | Mixed-methods (reports, national records, and surveys) | Caregivers of children | To review the epidemiology of polio acute flaccid paralysis surveillance, and the implementation of supplemental immunization activities in Afghanistan from 1997-2013 |
| 17 | The interactions of ethical notions and moral values of immediate stakeholders of immunisation services in two Indian states: a qualitative study | Joe Varghese, Raman Kutty, et al. | 2012 | India | Qualitative (interviews, focus group discussions, non-participant observation) | Providers, beneficiaries, mothers of under five children | Examines the existing norms regarding immunisation within the communities and the ethical notions that govern the actions of different health professionals and their collective synergistic or conflicting effects on the governance of the programme |
| 18 | Advancing the application of systems thinking in health: understanding the growing complexity governing immunization services in Kerala, India | Joe Varghese, Rama Kutty, et al. | 2014 | India | Qualitative (literature and document review, interviews, focus group discussions, non-participant observation | Immunization service providers, community health workers, mothers of children | Seeks to use a complex adaptive system lens to understand the features of a complex system that governs childhood immunization in parts of the Indian state of Kerala, where immunization coverage drastically declined after a period of high coverage |
| 19 | Cross border wild polio virus transmission in CORE Group Polio Project areas in Ethiopia | Filimona Bisrat, Legesse Kidanel, et al. | 2013 | Ethiopia | Qualitative (interviews, focus group discussions) | Community volunteers, health extension workers, program coordinators, managers | To assess polio immunization activities and risk of wild polio virus importation in CORE Group Polio Project international border areas of Ethiopia |
| 20 | Qualitative study of wild polio cases in high risk districts of Uttar Pradesh, India | Jugal Kishore, D Pagare, et al. | 2003 | India | Qualitative (survey) | Community members in 5 high risk districts with confirmed polio cases and 5 un-affected villages in the same districts | To determine the reasons for continued occurrence of wild polio cases and the perceptions of the community |
