# Supplementary material for: What are the barriers and facilitators to polio vaccination and eradication programs? A systematic review
Source: PLOS Glob Public Health. 2022 Nov 16;2(11):e0001283. doi: 10.1371/journal.pgph.0001283 (PMC10022167; doi:10.1371/journal.pgph.0001283)
Supplement: S1 Table — (DOCX) [file pgph.0001283.s004.docx]

***S1 Table: Barriers to Polio Vaccination and Eradication Programs***

|  | Barriers | Excerpts | Country | CFIR Construct (Domain) |
| --- | --- | --- | --- | --- |
| 1 | Medical practitioners’ opposition against repeated rounds of polio | Several private medical practitioners across Kerala opposed the repeated rounds of polio campaign and advised their clients against vaccination as they thought it unnecessary for children in Kerala. (p#6)  (Varghese et al., 2012) | India | (Compatibility) - Inner Setting |
| 2 | Propaganda and Mistrust Against Vaccines and its efficacy | It is important to consider the perception of parents who did not vaccinate their children in the context of widespread propaganda against vaccination programmes in Kozhikode district. (pg 7)  (Varghese et al., 2012) | India | (Knowledge & Beliefs about the Intervention) - Characteristic of Individuals |
|  |  | Three children had not received a single dose of OPV because of the family perception that (i) polio drops are adulterated; (ii) this is a ruse by the government to decrease the population; (iii) it may cause infertility; and (iv) it may cause some harm to their children. (pg # 3)  (Kishore et al., 2003) | India |  |
|  |  | Most parents wanted to immunize their children but were not convinced of the efficacy of OPV, as some children had developed polio despite immunization. (pg # 4)  (Kishore et al., 2003) | India |  |
| 3 | Lack of access to healthcare | Lack of access to health care for the majority of people is a barrier to routine immunizations and AFP surveillance. Under these circumstances, there are fewer opportunities for interfaces between health care staff and children who are at risk for polio (pg # 93)  (Ndiaye et al., 2003) | Niger | (Available Resources) -Inner setting |
| 4 | Cultural Beliefs | Our interviews with women whom we met in waiting rooms in health centers confirmed the importance of cultural beliefs. The period from when parents start seeing a local healer to when they realize that there is no improvement and therefore decide to go to the health center is 2–4 weeks, and hence the detection, reporting and investigation of AFP cases are either prevented or delayed. (93)  (Ndiaye et al., 2003) | Niger | (Knowledge & Beliefs about the Intervention) - Characteristics of Individuals |
| 5 | Poor Financial, Organizational, logistical, and OPV Quality Constraints | We repeatedly heard from parents, workers, and LGA-level officials that the mismatch between poor-quality health services generally and frequent campaigns was the driving force behind refusals. (pg # 12)  (Closser et al., 2016) | Nepal, India, Pakistan, Ethiopia, Nigeria, Rwanda, Angola | (Available Resources) - Inner Setting |
|  |  | All health extension workers stated that polio immunization coverage was low along the international border areas. The possible reasons for the low immunization coverage included shortage of vaccine and supplies, non-functionality of refrigerators, lack of training, cancellation of immunization sessions, and unavailability of health posts to deliver the services. (pg #33)  (Bisrat et al., 2013) | Ethiopia |  |
| 6 | Presence of Rumours | The presence of rumors which discouraged women and families from having their children vaccinated was acknowledged […] The rumors included inducing sterility in women following vaccination and disability in children who are vaccinated.  (pg. 36)  (Bisrat et al., 2013) | Ethiopia | (Knowledge & Beliefs about the Intervention) -Characteristics of Individuals |
| 7 | Suspicions of immunization  Side Effects | An instance was mentioned where a healthcare provider had to sign an agreement with women declaring that vaccination would not cause sterility. Actual and perceived side effects were another reason for avoiding vaccination (pg. 36).  Health extension workers in Lariee woreda indicated that one of the reasons for not vaccinating children was fear of side effects from injectable vaccines.  (pg # 33)  (Bisrat et al., 2013) | Ethiopia | (Knowledge & Beliefs about the Intervention) - Characteristics of Individuals |
|  |  | Lack of information, fear of side effects and trust in the programme were among the reasons cited for non-acceptors. (3325)  “Vaccine maybe harmful” was also mentioned by a few respondents. (3325) (Murele et al., 2014) | Nigeria |  |
|  |  | Intensification of immunization, especially the house-to-house rounds, had led certain sections of the community to question the motive of the government and strengthened their suspicion that the vaccine caused infertility (pg #4)  (Kishore et al., 2003) | India |  |
|  |  | Most of them said that there was resistance among the population in some areas due to the fear that OPV causes infertility. (pg # 4)  They were also aware of the rumour of OPV causing infertility. One of them stated that certain groups had distributed pamphlets directing people not to immunize their children with OPV as it may be harmful. (pg # 5)  (Kishore et al., 2003) | India |  |
|  |  | This mistrust was also seen with the politics of fertility […] They believed the polio program was simply a “reincarnation,” or a new manifestation, of the ‘Family Planning Program.’ When asked if they thought the sterilization rumors associated with the polio program were linked to this history, they responded: Respondent: Yes, from that time which is why we are being attacked by polio (program) [sic]  (pg # 15)  (Hussain et al., 2015) | India |  |
|  |  | Most reasons cited for refusal were based on the misconception that polio vaccination was associated with sterility (Pishin 48.6 per cent, Bajaur 66.7per cent, and Karachi 36.2per cent). (pg # 27)  (Habib et al., 2017) | Pakistan |  |
|  |  | As we said earlier, low education is the main problem. It makes people think that there may be some chemical in the OPV that cause infertility among children (FG 1: HW 5, 6). (p # 4)  (Khan et al., 2016) | Pakistan |  |
|  |  | Some people still believe in conspiracy theories of hidden agendas of America and the Central Intelligence Agency (CIA) to make their children sterile. (pg #4)  (Khan et al., 2016) | Pakistan |  |
|  |  | For instance, one participant noted that he had to overcome his wife’s hesitancy to get their children vaccinated after his wife had heard rumors, spread through media, that polio vaccine had caused deaths of children: ‘‘My wife was so worried. She did not want to vaccinate our children.”(pg # 3698)  (Shah et al., 2019) | Pakistan |  |
| 8 | Relocation of mothers | Furthermore, it was indicated that mothers move from place to place with their children making it difficult to vaccinate their children on time. (pg # 33)  Almost all the woreda health office respondents agreed that people are vulnerable to cross-border polio transmission because there is free movement of people from neighboring countries to Ethiopia in these woredas and vice versa. (pg # 34)  (Bisrat et al., 2013) | Ethiopia | N/A |
|  |  | Some people accepted that they had migrated to other states to get seasonal jobs, and therefore their children had missed the OPV doses during the ‘mop-up’ rounds. (pg # 4)  (Kishore et al., 2003) | India |  |
| 9 | Mothers avoiding OPV | Some women ran away with their children to the farms to avoid giving them OPV. (pg # 5)  (Kishore et al., 2003) | India | (Other Personal Attributes)   -Characteristics of Individuals |
| 10 | Lack of information, Ignorance, and Illiteracy | Among the remaining 191 children […] lack of awareness (24%) […] were the main reasons for children not being vaccinated.  (pg # 1298)  (Pervaiz et al., 2017) | Pakistan | (Knowledge & Beliefs about the Intervention) - Characteristics of Individuals - |
|  |  | Lack of information, fear of side effects and trust in the programmed were among the reasons cited for non-acceptors. (3325)  Small proportions (4% and 6%) of the acceptors and non-acceptors respectively had post-secondary education, while 17% and 23% of the acceptors and non-acceptors had no formal education. Seventeen percent (17) of the acceptors had secondary education compared with 3% of the non-acceptors had secondary education. Those with primary education constituted 5% and 10% of the acceptors and non-acceptors respectively (pg # 3325)  (Murele et al., 2014) | Nigeria |  |
|  |  | The refusal respondents view barriers to immunization as ignorance, no felt need, lack of basic necessities e.g. drugs in hospitals and water service.  On knowledge on if immunization prevents diseases: More than half of urban males answered ‘no’ compared to few of rural males.(pg # 3325)  The refusal respondents were asked to mention best ways to protect against polio virus. Majority of the respondents mentioned drinking of clean water as a way to avoid polio virus. Others identified ways to protect against polio included proper care and belief in God. Urban and rural female respondents mentioned proper care as a way of protection. (pg # 3326)  (Murele et al., 2014) | Nigeria |  |
|  |  | It was observed that there were 15 cases who did not receive pulse-polio dose on 23 January 2000. The reasons were negligence, fear, and unawareness. Some people got fed-up of repeated polio doses. Majority of the informants were females (pg # 648)  (Chincholikar et al., 2000) | India |  |
|  |  | None of the groups were aware of the need for additional doses of OPV. Some felt that the government had intensified the immunization drive because of the increase in polio cases. Most villagers were not aware of the cause and mode of transmission of the poliovirus. (pg # 4)  (Kishore et al., 2003) | India |  |
|  |  | Eighty-one children in 51 families did not receive oral vaccine[…] 20 per cent, misunderstanding or insufficient information about the program.  (pg # 471 - 472)                                                                         (Cohart et al., 1962) | United States of America |  |
|  |  | During FGDs, community participants identified poor communication and mobilization strategies, poor performance of polio staff, family planning misperception, lack of awareness, and carelessness of government, society, and parents as the main reasons for continued virus circulation in the area.  (pg 27)                                                                          (Habib et al., 2017) | Pakistan |  |
|  |  | A low education level makes people think negatively and make excuses; in addition, such people can easily be influenced by the thoughts of those who campaign against polio (HW 5, 6).  (pg # 4)  (Khan et al., 2016) | Pakistan |  |
| 12 | Fear of illness of children or parents | Among the remaining 191 children, refusals (27%) […] Refusals were driven mostly by fear or illness of the child at the time of the campaign, but they only constituted approximately 2% of the 2,100 children assessed during the survey. (pg # 1298)                                                                       (Pervaiz et al., 2017) | Pakistan | (Other Personal Attributes) - Characteristics of Individuals |
|  |  | Twenty-nine per cent of these families gave illness of the child or parent as the reason for their children's failure to participate in the program (pg # 471-472)  (Cohart et al., 1962) | United States of America |  |
|  |  | Consequently, many of the families were resistant because they were afraid to vaccinate their already weakened children. Such families were labeled “XS,” that is, resistant due to sickness     (pg # 14)  (Hussain et al., 2015) | India |  |
| 13 | Unavailability of Children | Among the remaining 191 children […] absence of the child during the campaign (16%) were the main reasons for children not being vaccinated. (pg # 1298)                                                                       (Pervaiz et al., 2017) | Pakistan | (Available Resources) - Inner Setting |
|  |  | The data also documented that the majority (62%–100%) of children in these provinces were missed because they were out- side the house, or because children were sick, newborn, or sleeping when the vaccinators arrived. (S168)                                                                      (Simpson et al., 2014) | Afghanistan |  |
|  |  | Most people felt that the house-to-house rounds were good except in some villages in Moradabad and Bijnor, where the people complained that some children were missed by the vaccinators. (pg # 4)  (Kishore et al., 2003) | India |  |
|  |  | In Pishin,37per cent of households refused to vaccinate their children. The most frequent reason given in Bajaur, where 38 per cent children were not vaccinated with OPV, was the unavailability of child at home during polio vaccinator visit. (pg # 29)  (Habib et al., 2017) | Pakistan |  |
| 14 | Belief that vaccination was unnecessary | The refusal respondents view barriers to immunization as ignorance, no felt need, lack of basic necessities e.g. drugs in hospitals and water service […] For refusal respondents, when asked to mention reasons, why child was not vaccinated during the most recent campaign. “Parent did not want” vaccine were cited most frequently as an indicator of refusal (pg3325)                                                                  (Murele et al., 2014) | Nigeria | (Knowledge & Beliefs about the Intervention) -Characteristics of Individuals |
|  |  | Overt refusals accounted for < 3% of children not vaccinated, except for the southern provinces where overt refusals accounted for up to 9.4% of missed children. (S168)                                                                       (Simpson et al., 2014) | Afghanistan |  |
|  |  | Eighty-one children in 51 families did not receive oral vaccine […] 12 percent felt oral vaccination was unnecessary (pg # 471-472)  (Cohart et al., 1962) | United States of America |  |
|  |  | That resistance caused a heated debate between the vaccination personnel and the women in the household. When the vaccinators found children in the household, the women claimed that all were more than five years old. At last, the team left for the next household without vaccinating any children. (pg # 11)  (Closser et al., 2016) | Nepal, India, Pakistan, Ethiopia, Nigeria, Rwanda, Angola |  |
| 15 | Lack of basic necessities | The refusal respondents view barriers to immunization as ignorance, no felt need, lack of basic necessities e.g., drugs in hospitals and water service. (pg # 3325)                                                                      (Murele et al., 2014) | Nigeria | (Patient Needs and Resources) - Outer Setting |
| 16 | Inaccessibility to reach children | District-level PCAs demonstrated pockets of missed children […] 11% of targeted children were missed, with 2% due to inaccessibility. (S168) […] >5% of children were missed during SIAs because of inaccessibility, and an additional 15 districts reported 1%– 4.9% missed children for the same reason. (pg # S169)  The mobility of staff during SIAs seriously compromised because of control non-governmental forces. In the LPD’s of the South region 5% to 6% of targeted children are reported as missed during each SIA because of Inaccessibility (pg 169)                                                                        (Simpson et al., 2014) | Afghanistan | N/A |
| 17 | Absence or Failure of vaccination teams visiting homes | Also, in the south and in 1 western province, the PCA data showed that the failure of vaccination teams visiting homes accounted for 7.6%–56.6% of missed children.  (S168)                                                     (Simpson et al., 2014) | Afghanistan | (Design quality and packaging) - Intervention characteristics |
|  |  | In Karachi 29 per cent children did not receive OPV due to absence of polio vaccination teams from their duty (pg # 29)                                         (Habib et al., 2017) | Pakistan |  |
| 18 | Lack of Infrastructure | Weak SIA implementation was also the main problem in parts of the endemic areas of northern India, such as in the hard-to-reach flood plains of the Kosi river basin in central Bihar state, where the very weak health infrastructure was at the root of the continued WPV transmission. (pg # D81) (Aylward et al., 2011) | Global | (Available Resources) - Inner Setting |
|  |  | Additional resistance stemmed from the fact that families felt the lack of infrastructure allowed the spread of disease, including polio. (pg 13)                                                                          (Hussain et al., 2015) | India |  |
| 19 | Inconvenience to vaccine access | Eighty-one children in 51 families did not receive oral vaccine […]18 percent, inconvenience, such as difficult clinic hours or lack of transportation (pg # 471-472) (Cohart et al., 1962) | United States of America | (Available Resources) – Inner Setting |
| 20 | Fear of vaccination being experimental | Eighty-one children in 51 families did not receive oral vaccine […] 16 per cent were afraid of it because they felt it was experimental.(pg # 471-472)  (Cohart et al., 1962) | United States of America | (Knowledge and Beliefs about the Intervention - Characteristics of Individuals |
| 21 | Protests by Alternative Medicine Providers | In northern Kerala many homeopathic practitioners have actively discouraged their clients from immunizing their children. Several study respondents believed that the strong influence of homeopathic medicine practitioners on house- holds in northern Kerala helped convincing them against immunization. (pg # 7)  (Varghese et al., 2014) | India | (External Change Agents) - Process |
| 22 | Anti-Immunization Debates | In the context of declining acceptability of the polio campaign, the debates that challenged the immunization programs received further credibility […] Although these frequent debates were centered on IPPI, they began to influence the community’s trust in vaccines. (pg # 7)  (Varghese et al., 2014) | India | (Evidence Strength and Quality) – Intervention Characteristics |
| 23 | Vaccine-resistant areas | It was also observed that the resistance against vaccination was often limited to geographical locations. It was observed during the house visits in vaccine-resistant areas of Kozhikode district that most of the unvaccinated children are found in households of close geographical vicinities. (pg4)                                                                        (Varghese et al., 2014) | India | (Knowledge and beliefs about the Intervention - Characteristics of Individuals |
| 24 | health worker and opinion leaders’ recommendations | In the FGDs it was said that some health workers and opinion leaders do not vaccinate their children and as a result the rest of the lay people tend to emulate them by also refusing to vaccinate (pg # 367)  (Nuwaha et al., 2000) | Uganda | (Compatibility) - Inner setting & Opinion leaders (Process) |
| 25 | Government distrust | Though the primary causes for social “resistance” to vaccination were regarding development and illness, some continued to be based on distrust of the local government…At the time of this study, people who feared the vaccine might sterilize their children in Aligarh were more likely to blame the government, polio workers, or flaws with the vaccine rather than an American or Western plot against Muslims     (pg # 14)  (Hussain et al., 2015) | India | (Knowledge and beliefs about intervention) - Individual characteristics |
| 26 | Governmental Distrust due to Neglect of Muslim Slums | Respondents felt that the neglect of Muslim slums facilitated further distrust of the government and corresponding resistance to vaccination. (pg # 12)   (Hussain et al., 2015) | India | (Knowledge and beliefs about intervention) - I**ndividual characteristics** |
| 27 | Resistance due to the lack of basic health facilities | All the participants regarded the lack of basic health facilities as the primary cause of mistrust, and consequently of the resistance to OPV, in some Pakhtun communities. (pg # 3700)  (Shah et al., 2019) | Pakistan | (Knowledge and beliefs about intervention) - Individual characteristics |
| 28 | Safety and Religious Misconceptions Regarding Vaccine | This misconception about sterility was compounded by other misconceptions, including: the vaccine is not ‘halal’ or impermissible under Islamic Law (highest in Bajaur25per cent), vaccine is not safe (highest in Karachi 19 per cent), and vaccine contradicted religious beliefs (highest in Bajaur16 per cent).   (pg # 27)  (Habib et al., 2017) | Pakistan | (Knowledge and Beliefs about the Intervention) - Characteristics of Individuals |
|  |  | Witchcraft accusations, rooted in local politics, were clearly a locally specific way of expressing vaccine distrust in Kumbotso.  (pg # 12)  (Closser et al., 2016) | Nepal, India, Pakistan, Ethiopia, Nigeria, Rwanda, Angola |  |
| 29 | Accepting American Vaccination Erodes Muslim Credentials | In SITE Town, Pakistan, a community with large numbers of drone refugees, some people argued that accepting “American” vaccination would erode one’s Muslim credentials Kalimah. (Pg. 336)  (Closser et al., 2016) | Nepal, India, Pakistan, Ethiopia, Nigeria, Rwanda, Angola | (Knowledge and Beliefs about the Intervention) - Characteristics of Individuals |
| 30 | Fear of vaccines due to past Ebola experience | A small number of participants recounted how immunization teams (during the PIRIs) had worn personal protective equipment (PPE) and that this caused communities to be afraid. (pg. 86-87).  Before Ebola we took the measles vaccine, but the reason we didn’t take it now was because of Ebola. When the people came to give the vaccine, they were bringing those Ebola things, like chlorine and buckets and temperature scanners, those things from Ebola time. (pg. 86-87).  (Bedford et al., 2017) | Liberia | (Knowledge and beliefs about the intervention) - Characteristics of Individuals |
|  |  | Similarly, a community leader in Monrovia, who had been a member of his community’s task force during Ebola, explained, “For the campaign, people had the notion that they were coming for the children, to kill the children with the immunizations.” (pg. 87)  (Bedford et al., 2017) | Liberia |  |
| 31 | Participant perception of advanced age | People had failed to receive vaccinations, and so we asked the respondent why each nonvaccinated  member of the household had not been vaccinated. The most frequent reason was “too old" which, if combined with the similar response of "vaccination is not necessary for adults," accounted for about 45 percent of all reasons given in both counties (pg # 553)  (Ianni et al., 1960) | United States of America | (Knowledge and beliefs about the intervention) - Characteristics of Individuals |
| 32 | Lax or Disinterest of Parents concerning vaccinations | In a separate question we asked the respondent's opinion as to why most teenagers had not been vaccinated. Here the laxity was assigned to the parents; in about 25 percent the cases in both counties the response was that parents were too lax or disinterested  (pg # 554)  (Ianni et al., 1960) | United States of America | (Knowledge and beliefs about the intervention) - Characteristics of Individuals |
| 33 | Conflict and security issues | The participants explained how, in the wake of 9/11, the global war on terror and the geopolitical situation of Pakistan and Afghanistan impacted polio eradication and other immunization efforts in the country. (pg # 7) (Haq et al., 2019) | Pakistan | N/A |
|  |  | Other challenges in this area are the security issues (FG 3: HW 8–10 & FG 4: HW 2–5). Due to the recent terrorist attacks we sometimes have to look over our shoulders to check if someone is following us (FG 3: HW 8). Due to attacks on polio teams we sometimes have to be careful (pg 2077) (Khan et al., 2016) | Pakistan |  |
| 34 | Frequent visits | Some people refuse to have their child vaccinated if he or she has been vaccinated in last 3–4 months (FG 4: HW 1–3). (pg # 4)  (Khan et al., 2016) | Pakistan | (Design Quality & Packaging) - (Intervention characteristics) |
| 35 | Refusal as a means of protest | Therefore, some parents refused to vaccinate their children as a form of political leverage to get the government to address their political/economic problems. (pg # 3700)  (Shah et al., 2019) | Pakistan | (Knowledge and beliefs about intervention) -  characteristics of individuals |
| 36 | Reliance on Religious Leaders that oppose OPV | The participants described a complex relationship between religious beliefs and OPV skepticism and resistance as barriers to OPV. (pg # 3700) (Shah et al., 2019) | Pakistan | (Knowledge and beliefs about intervention) - characteristics of individuals & (External change agents) - Process |
|  |  | He explained that a combination of low literacy among these communities and mistrust in international and governmental organizations creates reliance on religious leaders who oppose OPV. (pg # 3700)  (Shah et al., 2019) | Pakistan |  |
