# Supplementary material for: What are the barriers and facilitators to polio vaccination and eradication programs? A systematic review
Source: PLOS Glob Public Health. 2022 Nov 16;2(11):e0001283. doi: 10.1371/journal.pgph.0001283 (PMC10022167; doi:10.1371/journal.pgph.0001283)
Supplement: S2 Table — (DOCX) [file pgph.0001283.s005.docx]

***S2 Table: Facilitators to Polio Vaccination and Eradication Programs***

|  | **Facilitators** | **Excerpts** | **Country** | **CFIR Construct (Domain)** |
| --- | --- | --- | --- | --- |
| 1. | Parental Moral Obligation and Inability to be deviant | It is widespread in societies which have a good coverage of immunization. In such areas, vaccines have become a societal norm making it difficult for parents to avoid it. The FGDs with mothers held in areas of high vaccination coverage reiterated that in an environment where all parents vaccinated their children, it was difficult to be a deviant. (pg # 6)  (Varghese et al., 2012) | India | (Knowledge and beliefs about the intervention) - Characteristics of Individuals |
|  |  | Parents’ moral obligation towards their children plays another major role in guiding the immunization programme and contributes to its sustainability. This value comes out of the parent’s feeling that immunization is their duty towards children. (pg # 7)  (Varghese et al., 2012) | India |  |
| 2. | Coercive Means | The overwhelming emphasis on coverage results in the use of coercive means to achieve targets. It restricts the options for refusal to undergo immunization or for postponement of immunization available to beneficiaries. (pg # 5)  (Varghese et al., 2012) | India | (Tension for change) - Inner Setting |
|  |  | A majority of the acceptors presented their children for vaccination because they have been told to do so ]…] The benefits of vaccination were acknowledged by the rural health workers. (pg # 3325)  (Murele et al., 2014) | Nigeria |  |
| 3. | Fear of Contracting the Disease | they are willing to take their children for vaccination at local clinics and hospitals when diseases such as measles or meningitis threaten. At such times, their fear of disease overshadows the perceived risks of vaccination (pg # 3324)  (Murele et al., 2014) | Nigeria | (Knowledge and beliefs about the intervention) - Characteristics of Individuals |
| 4. | Good social mobilization system | Mobilizers who visit homes prior to SIAs are present in >90% of targeted low-performing communities in the south. These mobilizers identify all eligible children and mark their homes so that vaccinators know how many children in a household need vaccine, decreasing the likelihood of missed children.  (S170)                                                                (Simpson et al., 2014) | Afghanistan | (Available resources) - Inner Setting |
| 5. | Finding and Mapping Marginalized Population | In most of our case studies, people working on polio eradication found, mapped, and repeatedly visited populations that were previously unreached by other health services, increasing the amount of vaccination. (pg. 508)                                                                                       (Closser et al., 2014) | 7 countries in South Asia and Sub-Saharan  Africa, Ethiopia, Nigeria, Rwanda, Angola, India, Nepal, Pakistan | (Available resources) - Inner Setting |
| 7. | Improvements in infrastructure | These problems in central Bihar were solved only after a massive increase in staff and technical support, linked to improvements in infrastructure, facilitating significant improvements in coverage during SIAs (pg # D81) (Aylward et al., 2011) | Global | (Available resources) - Inner Setting |
| 8. | Belief in the vaccine’s ability to protect against polio | Regardless of social class, a high proportion of families whose children received oral vaccine gave as their reason for participation in the program the added protection that the vaccine would afford (pg # 468)   (Cohart et al., 1962) | United States of America | (Knowledge and beliefs about the intervention) - Characteristics of Individuals |
|  |  | Initial opposition was neutralized by the public’s confidence in vaccination in general and considerable state support for the program. For example, according to our respondents, there was extensive participation in the program implementation by several actors, including several government departments, in addition to health, as well as Panchayats (village level elected governance institution), NGOs, and schools. (pg # 5)                                                                                       (Varghese et al., 2014) | India |  |
|  |  | As to reasons for immunization, all six FGDs said that the major aim was to ‘weaken’ the diseases and or ‘strengthen’ the children’s capability in fighting diseases. (pg # 367)   (Nuwaha et al., 2000) | Uganda |  |
|  |  | The participants stated that they were supportive of the OPV because they have seen the risks associated with contracting polio- virus and the benefits of its prevention through vaccine. (pg # 3697)                                (Shah et al., 2019) | Pakistan |  |
| 9. | Influence from health-focused champions | Roughly one-third of these families stated that a physician had influenced their decision, while slightly more than one-quarter said that their decision was influenced by the interviewer.  (pg # 468 469)  (Cohart et al., 1962) | United States of America | (Champions) - Process |
|  |  | The introduction of community health workers known as Accredited Social Health Activists (ASHAs) has, therefore, played a positive influence in decision making of the parents on immunization. ASHA’s status as local women known to the other members of the community gave her special advantage in influencing the perceptions of com- munity on immunization issues. (pg # 9)                                                                                              (Varghese et al., 2014) | India |  |
|  |  | Ground-level workers also cited the engagement of political and other leaders as reasons for increased acceptance of polio vaccine. (pg # 12) (Closser et al., 2016) | Nepal, India, Pakistan, Ethiopia, Nigeria, Rwanda, Angola |  |
|  |  | A second program relevant to increased acceptance of vaccines is the Anganwadi program…The same Anganwadi workers also go door-to-door providing polio vaccine during scheduled campaigns. So, vaccine is being provided to children by the same trusted women who teach and feed them.  (pg # 15)                                                                                                (Closser et al., 2016) | Nepal, India, Pakistan, Ethiopia, Nigeria, Rwanda, Angola |  |
| 10. | Increased Accessibility to immunization due to close NID Posts | All FGDs reported that unlike for routine immunization, NIDs posts were very near and within a short walking distance. NIDs vaccinators were said to be friendly and not to abuse mothers as was sometimes the case during routine vaccinations. (pg # 367)  (Nuwaha et al., 2000) | Uganda | (Available resources) - Inner Setting |
| 11. | Knowledge/Awareness of Polio Vaccines | When specifically asked about the chances of getting polio if vaccine is not given,59 per cent from Pishin, 78per cent from Bajaur, and 81per cent from Karachi knew that oral polio vaccine (OPV) is necessary for preventing polio. (pg # 27)  (Habib et al., 2017) | Pakistan | (Knowledge and beliefs about the intervention) - Characteristics of Individuals |
|  |  | As a ground-level worker explained: Earlier, when we used to go door-to-door for polio campaigns, people would say they didn’t want to give their children the vaccine. My children will get a fever, people would say, or they’d say the vaccine would sterilize their children. . . . They used to hide their children. But now, the public is aware. People come themselves to get vaccination (pg # 15)  (Closser et al., 2016) | Nepal, India, Pakistan, Ethiopia, Nigeria, Rwanda, Angola |  |
|  |  | “We don’t worry about the measles and polio vaccine because we know it from before, from when the children were younger.”  One of the most dominant was knowledge and experience of accepting measles and polio vaccines prior to Ebola. (pg. 87-88)  (Bedford et al., 2017) | Liberia |  |
| 12. | Multiple polio campaigns | In case studies with two or fewer campaigns per year, and one with four campaigns per year, respondents asserted that people were happy with polio campaigns, largely because they no longer had to fear polio. In these districts, refusals were rare. (pg # 9)  (Closser et al., 2016) | Nepal, India, Pakistan, Ethiopia, Nigeria, Rwanda, Angola | (Design quality and packaging) - intervention characteristics |
| 14. | Increased Trust in Government Health Services | These improvements led to increased trust in government health services. “Trust has increased very much,” a frontline worker said. One manifestation of this trust was acceptance of vaccination. Our respondents were nearly unanimous in asserting that there has been a sea change in public acceptance of all vaccines, including polio vaccines, over the last 15 years. People used to fear vaccination, respondents told us, but now they largely accept it. (pg # 15)  (Closser et al., 2016) | Nepal, India, Pakistan, Ethiopia, Nigeria, Rwanda, Angola | (Knowledge and beliefs about intervention) - characteristics of individuals |
|  | Involvement of religious scholars | Participants noted that the involvement of religious scholars in the OPV campaigns also helped the cause of polio eradication… “The involvement of the religious scholars has decreased the number of non-compliant parents.”  (pg # 3701)                                                                                         (Shah et al., 2019) | Pakistan | (Champions) - Process |
|  |  | The participants described a decrease in religious resistance to OPV. They attributed this decrease to the defeat of militants and to the involvement of religious scholars in polio vaccination campaigns since 2014. (pg # 3701)                                                                                        (Shah et al., 2019) |  |  |
| 15. | Government mandating polio vaccination to fly abroad | This participant was referring to the fact that the Pakistani government mandated presenting polio vaccination certificates to immigration officers at airports before flying abroad. (pg # 3697)  (Shah et al., 2019) | Pakistan | (External Policies and Incentives) - Outer Setting |
| 16. | Decrease in religious resistance to OPV | The participants described a decrease in religious resistance to OPV. They attributed this decrease to the defeat of militants and to the involvement of religious scholars in polio vaccination campaigns since 2014. (pg # 3701)                                                                                (Shah et al., 2019) | Pakistan | (Knowledge and beliefs about intervention) - characteristics of individuals  & (Champions) - Process |
