# Supplementary material for: What are the barriers and facilitators to polio vaccination and eradication programs? A systematic review
Source: PLOS Glob Public Health. 2022 Nov 16;2(11):e0001283. doi: 10.1371/journal.pgph.0001283 (PMC10022167; doi:10.1371/journal.pgph.0001283)
Supplement: Attachment — Submitted filename: Reviewers Response Document_Sept15.docx [file pgph.0001283.s007.docx]

# Review Comments to the Author

**Reviewer #1:** This research was done using appropriate methodological techniques and rigor according to recognised criteria and templates for qualitative reviews. The analysis of the qualitative findings was done excellently well using the CFIR framework and as such, statistical analysis was not applicable. It was well written not requiring further editing.

It was interesting to see this paper, a more global multi-context review aside the available reviews limited to a region (e.g SSA). This provided a richer evidence base and data though some of the findings were not entirely new because the challenge with Polio in recent years has been limited to certain LMIC which the review still captured. I was particularly happy to see the health system of Rwanda in the discussion.

I was also happy to see the CFIR approach used to analyse the findings. However, there was no statement on the comparison of this approach with the best-fit framework which I had looked forward to reading. It will ne good to make a methodological statement on this bearing in mind that previous studies that can be compared with were done in different times, restricted to certain regions.

Thank you for your comment. We had planned to compare this approach with the best-fit framework. However, we later decided not to undertake this step due to its complexity but forgot to remove that statement from the introduction. The statement referring to the best-fit framework has now been taken out.

It will be good for the authors to add a section on the strengths of this review in addition to the limitations. In reviewing these two sections (strengths and limitations), take into account the ongoing discussions of how multi-context reviews should be used compared to single-context reviews. This is critical and will provide insights into how the findings should be used or utilised by policymakers and practitioners. Moreso, your review was global, highly heterogenous in nature comprising both high income countries and LMIC. kindly refer to this for more information (Booth A, Mshelia S, Analo CV, Nyakang'o SB. Qualitative evidence syntheses: Assessing the relative contributions of multi‐context and single‐context reviews. Journal of Advanced Nursing. 2019 Dec;75(12):3812-22 ) assessed here <https://doi.org/10.1111/jan.14186>.

Welldone to the authors for a good job.

Thank you for your comment. We agree and have included a section on the strengths.

*This review employed the multi-context approach to synthesize data, providing a wide range of evidence that allows cross-setting comparison and applying study findings to broader settings.^48,49^ Although such an approach offers transferable patterns of findings at a global level, they may be too general to target important local characteristics or audiences, leading to the study overlooking the relevance of specific contexts and blurring critical differences across different studies.^48,49^ It might also appear that the study is ignoring the significance of context in evidence synthesis.^49^ Nonetheless, barriers and facilitators associated with polio vaccination and eradication are not too varied. Thus, contextual heterogeneity is mostly minimal in such programs aside from outliers such as political unrest that impede vaccination in certain parts of the world.*

**Reviewer #2:** Reviewer Comments
This manuscript studied Barriers and Facilitators to Polio Vaccination and Eradication Programs: A Systematic Review. The paper is very interesting, but there are some minor issues. However, some other problems in the manuscript are still concerned in the following:
1)More keywords are suggested.

We have added more keywords:

***Keywords:*** *polio; poliomyelitis; vaccination; implementation; immunization; CFIR; consolidated framework for implementation research; eradiation; infectious disease*

2)Divide your abstract in different sections (i.e Background, objective, methods, results, conclusion and recommendations)

The abstract has been divided into different sections.

3)A section of study area and data is suggested.

The sections on participants and countries in the results section provide more context of the study areas of the eligible studies.

4)The language should be polished.

Thank you for your comment. We have made changes throughout the manuscript to improve flow and clarity

5)Authors use old references sometimes in the paper (eg reference number 3,4,5,12 and 15 etc). Please use the updated (2020-21-22) study or research.

Thank you for your comments. We have updated the references with newer references. However, It is important to note that our search strategy was executed in 2020, so studies from 2021-2022 would not have been captured in our study.
