# Supplementary material for: What are the barriers and facilitators to polio vaccination and eradication programs? A systematic review
Source: PLOS Glob Public Health. 2022 Nov 16;2(11):e0001283. doi: 10.1371/journal.pgph.0001283 (PMC10022167; doi:10.1371/journal.pgph.0001283)
Supplement: Attachment — Submitted filename: Polio Response to reviewer_Oct20.docx [file pgph.0001283.s008.docx]

1. Please update the Methods section to report the search key words (strings) used to collect your data in sufficient detail for another researcher to replicate the search.

- Thank you for your comment. The search key words (strings) used to collect the data are included in supplementary file 1. We have included the following sentence in the manuscript to direct readers to this file: “*The search strings used in each database are provided in Supplementary File 1*.”

2. Please review your reference list to ensure that it is complete and correct. If you have cited papers that have been retracted, please include the rationale for doing so in the manuscript text, or remove these references and replace them with relevant current references. Any changes to the reference list should be mentioned in the rebuttal letter that accompanies your revised manuscript. If you need to cite a retracted article, indicate the article’s retracted status in the References list and also include a citation and full reference for the retraction notice.

- Thank you for your comment. All of the references have been checked and none were retracted. Moreover, we have updated the references to ensure that they are complete and correct. Lastly, the reference list has been modified to reflect when they appear in the manuscript. The table below explains all the changes made to the reference list from the previous version (the middle column), to what it was changed to (the right column).

| **Study** | **Previous citation in manuscript/reference list** | **Revised citation in manuscript/reference list** |
| --- | --- | --- |
| (WHO, 2021) | 1. | 1. |
| (Mshelia et al., 2020) | 2. | 2. |
| (Akinyemi et al., 2021) | 3. | 3. |
| (Bellatin et al., 2021) | 4. | 4. |
| (Cintyamena et al., 2021) | 5. | 5. |
| (Jamal et al., 2020) | 6. | 6. |
| (Olateju et al., 2022) | 7. | 7. |
| (Ataullahjan et al., 2021) | 8. | 8. |
| (Alonge et al., 2020) | 9. | 9. |
| (Page et al., 2021) | 10. | 10. |
| (Ezezika, Aman, et al) | 11. | 11. |
| (Brice) | 12. | 12. |
| (MMAT, 2018) | 13. | 13. |
| (Bisrat et al., 2013) | 16. | 14. |
| (Murele et al., 2014) | 17. | 15. |
| (Khan & Sahibzada, 2016) | 18. | 16. |
| (Kishore et al., 2003) | 19. | 17. |
| (Hussain et al., 2015) | 20. | 18. |
| (Habib et al., 2017) | 21. | 19. |
| (Shah et al., 2019) | 22. | 20. |
| (Cohart et al., 1962) | 23, | 21. |
| (Bedford et al., 2017) | 24. | 22. |
| (Closser et al., 2016) | 25. | 23. |
| (Ndiaye et al., 2003) | 27. | 24. |
| (Simpson et al., 2014) | 26. | 25. |
| (Aylward & Tangermann, 2011) | 14. | 26. |
| (Pervaiz et al., 2017) | 28. | 27. |
| (Closser et al., 2014) | 30. | 28. |
| (Nuwaha et al., 2000) | 29. | 29. |
| (Varghese et al., 2014) | 31. | 30. |
| (Varghese et al., 2013) | 32. | 31. |
| (Chincholikar & Prayag, 2000) | 33. | 32. |
| (Ianni et al., 1960) | 34. | 33. |
| (Haq et al., 2019) | 35. | 34. |
| (Choudhary et al., Mar 2021) | 36. | 35. |
| (Choudhary et al., Jul 2021) | 37. | 36. |
| (Lewis et al., 2020) | 38. | 37. |
| (Malik et al., 2020) | 39. | 38. |
| (Deressa et al., 2020) | 40. | 39. |
| (Osaghae et al., 2022) | 41. | 40. |
| (Weisen et al., 2022) | 42. | 41. |
| (Hu et al.) | 43. | 42 |
| (Toppa, 2020) | 44. | 43. |
| (Britannica) | 45. | 44. |
| Means et al., 2020) | 46. | 45. |
| (Damschroder et al., 2022) | 47. | 46. |
| (Booth et al., 2019) | 48. | 47. |
| (Hannes & Harden, 2011) | 49. | 48. |
| (Nilson, 2015) | 50. | 49. |
| (Ezezika, Gong, et al., 2021) | 51. | 50. |
| (Ezezika, Ragunathan, et al., 2021) | 52. | 51. |
